# A trade-off between fungal biofilm formation and persistence in the host

**DOI:** 10.1371/journal.pbio.3004027

**Published:** 2026-09-29

**Authors:** Shannon Esher Righi

**Affiliations:** Department of Microbiology and Immunology, Tulane University School of Medicine, New Orleans, Louisiana, United States of America

## Abstract

Fungal biofilm formation has long been considered an important virulence trait. A new study on Candida parapsilosis outbreak isolates in PLOS Biology challenges this, finding that low biofilm-producing strains are better adapted for immune evasion and survival.

The human fungal pathogen *Candida parapsilosis* has been implicated in an alarming increase in hospital-associated outbreaks worldwide [1]. While antifungal resistance is a growing feature of outbreak isolates, other microbial traits driving long-term persistence have yet to be identified. A recent PLOS Biology study by Daneshnia and colleagues [2] tracks the emergence of a striking phenotype shared among a collection of global fluconazole-resistant outbreak isolates: low biofilm production. Biofilms are communities of microorganisms that adhere to one another and surfaces through the production of a sticky extracellular matrix made up of sugars, proteins, and other molecules. In addition to providing a means for adherence, these structures also serve as a physical barrier against stressors, shielding organisms within the biofilm architecture from antimicrobial and immune cell access. The new study by Daneshnia and colleagues, challenges the classic view of biofilms as promoting persistence and antifungal resistance and instead considers what advantages human-associated microbes may gain by trading in their biofilms.

Infections involving biofilms are notoriously difficult to treat, leading to an estimated global healthcare cost of over $40 billion annually attributed to fungal biofilms alone [3]. *Candida* species including *C. parapsilosis* are both human commensals and common pathogens causing a range of diseases from superficial mucosal infections to life-threatening invasive infections that often arise from their ability to colonize indwelling medical devices, such as central lines used for the delivery of intravenous fluids or medications. Because of this outsized role in device-related infections, biofilm regulation and development have been intensively studied in several *Candida* species, particularly *C. albicans*. Biofilm formation generally occurs in four phases: colonization or adherence to a surface; cell proliferation and the start of matrix production; maturation of the community structure; and finally dispersal or release of cells [3]. In *Candida* species, these stages are tightly controlled by a complex transcriptional network that has evolved relatively recently, as evidenced by the presence of many “young” lineage-specific genes [4]. In contrast to ancient and broadly conserved “old” genes that are present across many species, “young” genes are those present only in closely related species and represent newly evolved processes. Daneshnia and colleagues discovered that their low biofilm-producing *C. parapsilosis* isolates have undergone widespread transcriptional reprogramming resulting in the upregulation of evolutionarily “old” genes involved in core metabolic functions [2], indicating a reversion towards more ancient, conserved cellular processes. This finding led to an intriguing question: what advantages might biofilm attenuation confer?

Biofilm formation is an incredibly energetically costly process, requiring upregulation of specialized genetic networks, rapid cellular proliferation, and the production of numerous extracellular matrix components. By contrast, long-term fungal survival within the host requires a high level of metabolic flexibility, with the propensity for metabolic adaptation able to outweigh the loss of traditional virulence factors [5]. Daneshnia and colleagues, found that low biofilm-producing *C. parapsilosis* isolates grew well across a variety of both stress and non-stress conditions and routinely outcompeted biofilm-producing isolates, demonstrating a clear fitness advantage under in vitro conditions [2]. This fitness advantage over biofilm-producing isolates is similar to that observed in experimental bacterial populations*,* where extracellular matrix-producing strains can be outcompeted by infiltrating nonproducing strains that do not have to expend energy making matrix material [6]. The next step was to connect these in vitro growth advantages back to the host environment.

The fungal cell wall is a dynamic structure that serves as the first point of contact with the host. The cell wall polysaccharide β-glucan is a prominent pathogen-associated molecular pattern. To control immune recognition, β-glucan exposure is routinely masked by the outer fibrillar mannan cell wall layer through a process that is tightly controlled and highly responsive to nutritional signals [7]. Daneshnia and colleagues, found that low biofilm-producing *C. parapsilosis* isolates exhibited elevated β-glucan masking, as evidenced by increased mannan exposure and decreased β-glucan exposure [2]. As expected, this cell wall arrangement led to decreased phagocytosis and killing by innate immune cells compared to biofilm producers with higher levels of β-glucan exposure, suggesting that the low biofilm phenotype is better able to evade immune detection in vivo. Perhaps paradoxically, when the authors transcriptionally profiled infected macrophages, they found that low biofilm-producing isolates induced a heightened proinflammatory state [2]. However, this finding indicates that, in addition to evading immune detection, these low biofilm isolates can also persist in settings of inflammatory activation. This, in fact, complements their data demonstrating enhanced stress tolerance in vitro. As further evidence, they found that low biofilm-producing isolates had increased survival in vivo in a mouse model of candidiasis, particularly in immune cell-rich organs [2].

Altogether, Daneshnia and colleagues present evidence for an evolutionary trade-off between biofilm formation and increased fitness within host tissues (Fig 1). This dichotomy is reminiscent of what is seen in pathogenic *Salmonella* strains: biofilm formation is conserved among strains that can colonize the intestines of multiple host species and cause localized gastroenteritis, but lost in strains that are adapted and restricted to single hosts and cause systemic and invasive diseases [8]. Similar observations have been made for commensal and invasive *Escherichia coli* strains. *C. parapsilosis* has been isolated from a variety of environmental niches and is a frequent commensal colonizer of human skin, yet its incidence and disease severity have rapidly increased in recent years [9]. Could the development of these immunoevasive, low biofilm-producing isolates represent a shift towards a more invasive lifestyle for *C. parapsilosis*? Changes in selective pressure, i.e., towards immune avoidance and long-term persistence, are thought to have contributed to the loss of biofilm among host-restricted *Salmonella* strains, making them more fit for survival within specific host niches. Daneshnia and colleagues suggest a similar explanation, which points towards the host environment as the main driver of strain evolution during these outbreaks.

**Fig 1:**
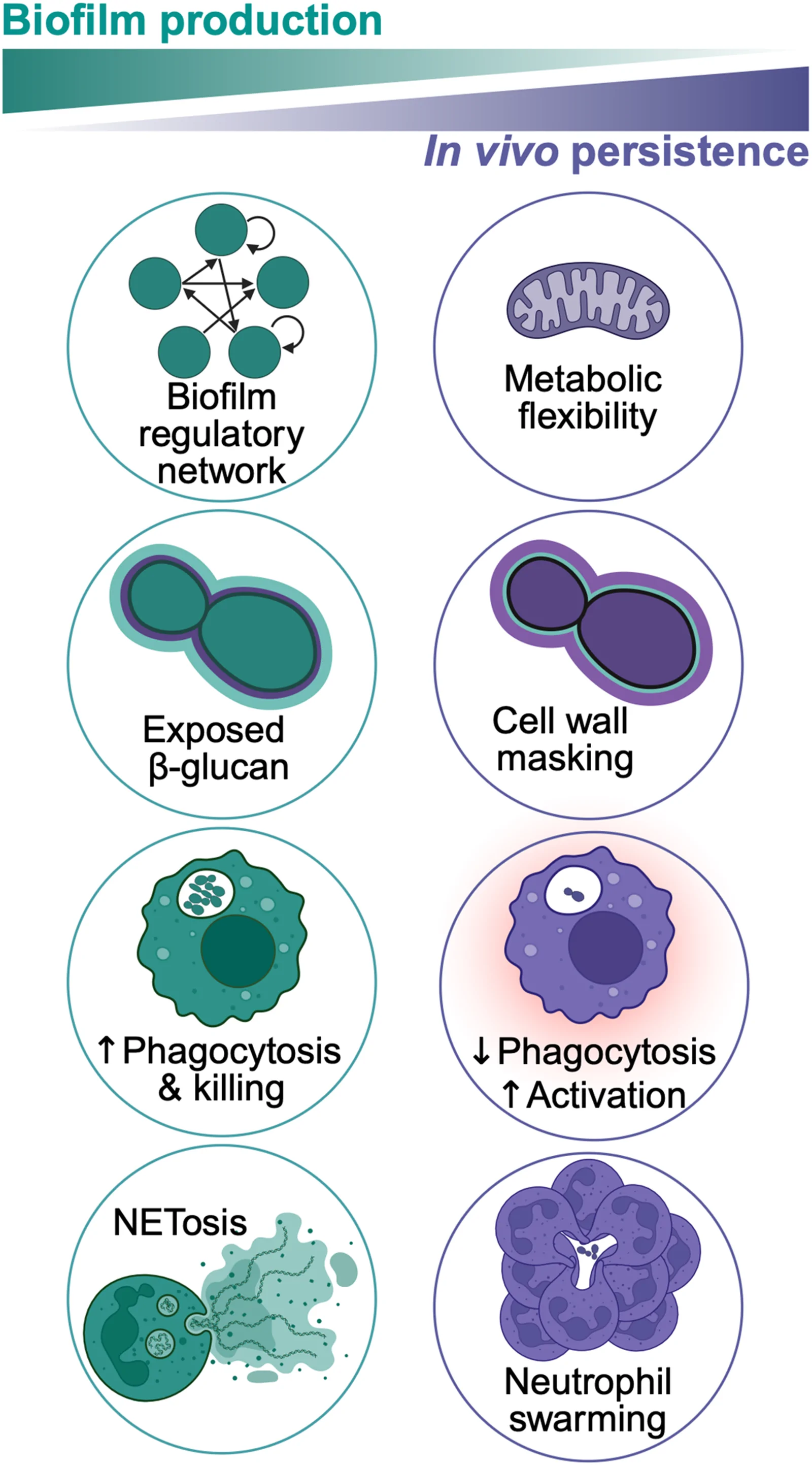
Trade-off between biofilm production and in vivo persistence. Biofilm production facilitates colonization of commensal organisms, promotes adherence, and confers intrinsic antifungal resistance. Biofilm-producing strains are under tight transcriptional control and expose β-glucan. This leads to increased phagocytosis and killing by macrophages, and extracellular NET production by neutrophils. By contrast, Daneshnia and colleagues, show that low biofilm-producing strains undergo transcriptional reprogramming to allow for metabolic flexibility. They mask their β-glucan to facilitate immune avoidance, leading to decreased phagocytosis by macrophages. However, when phagocytosed, low biofilm-producing strains are more fit for growth and survival within immune cell-rich niches of the host, leading to enhanced macrophage activation and neutrophil swarming. Together these features allow low biofilm-producing *C. parapsilosis* strains to survive and persist within the in vivo host environment. Created in BioRender. Righi, S (2026).

As with any exciting discovery, a number of outstanding questions remain. From a fungal genetics standpoint, the specific genes or mutations driving the low biofilm phenotype remain to be determined. Whether this phenotype and the underlying transcriptional rewiring are shared with other outbreak-associated *Candida* species also warrants further investigation. Furthermore, defining the connections between biofilm formation, metabolism, and cell wall biogenesis will provide novel and broadly applicable insights into fungal physiology. With respect to the enhanced immune evasion of these isolates, understanding how and why they simultaneously enhance macrophage activation and thrive in immune cell-rich organs is another avenue worth pursuing. Collectively, these open areas of investigation converge on a central question: is biofilm attenuation a cause or a consequence of host adaptation? The answer to that question is likely to have a significant impact on our evolving understanding of the host-pathogen interface.

In conclusion, this exciting study by Daneshnia and colleagues overturns our thinking about what traits may be beneficial to fungi trying to survive within the host. A thorough understanding of the driving forces behind the development of this low biofilm-producing phenotype may provide insight into the path from commensal to pathogen. From a therapeutic standpoint, more consideration may need to be given to the selective pressures imposed by drugs targeting traditional virulence factors such as biofilm formation. Finally, this work will continue to have important implications as we enter an age in which new fungal threats are expected to emerge due to a combination of changing environmental conditions and advances in medicine leading to ever-increasing numbers of immunosuppressed patients.
